# Mindfulness moments for clinicians in the midst of a pandemic

**DOI:** 10.1017/ipm.2020.59

**Published:** 2020-05-21

**Authors:** E. Hedderman, V. O’Doherty, S. O’Connor

**Affiliations:** 1 Lucena Clinic, Dublin, Ireland; 2 Department of Psychology, Tallaght University Hospital, Dublin, Ireland

**Keywords:** Clinicians, compassion, COVID-19, mindfulness, pandemic

## Abstract

Clinicians are routinely subjected to intense and stressful working environments, and the current COVID-19 crisis increases their risk of psychological distress. Mindfulness has been shown to improve life satisfaction, resilience to stress, self-compassion, compassion and general well-being in healthcare workers. Based on their clinical experience, the authors present mindfulness moments for clinicians (MMFC), a selection of short, simple and accessible mindfulness practices to promote resilience and compassion among clinicians working in this pandemic. The practices can be used on the job and are accessible to both novice and experienced meditators. Most of these practices are extracted from evidence-based mindfulness programmes. Further research is indicated to assess the effectiveness of using MMFC to support clinicians in their work and to promote resilience.

*Getting Grounded.*
*On your inbreath, gather your attention.*
*On your outbreath, drop into the body.*
*Shift your attention to the sensation of your feet on the floor.*
*Sense stability and support through this connection to the earth.*
*Rest in the experience of being grounded.* Joan Halifax (Halifax, ) 2020

## Introduction

Looking after the health of people provides many rewards but brings significant challenges. During a crisis, such as the current COVID-19 pandemic, these challenges can substantially increase. Healthcare workers are routinely subjected to intense stimuli in their work that may lead to burnout (Chernoff *et al*. 2018) and result in distress and emotional suffering (Ducar *et al*. 2020). In this current unprecedented global pandemic, front-line healthcare workers are risking their lives in the line of duty requiring them to demonstrate courage in the presence of fear.

A systematic Cochrane review (Ruotsalainen *et al*. 2014) reported that healthcare workers face high expectations, insufficient time allowance to fulfil those expectations, poor skills to manage stress-provoking stimuli and low social support at work when the stress response is triggered. This leads to symptoms of burnout, physical illness, an inability to provide high-quality healthcare services and increased cost to the individual and governing body due to sick leave and high staff turnover rates.

During the Ebola outbreak in 2014, front-line workers were also coping with an imminent threat to their lives, working excessive hours, fear of contamination for themselves and loved ones, the breakdown of social support systems, the deaths of colleagues and increased psychological distress (Lehmann *et al*. 2015). They were also at risk of developing psychological symptoms such as obsession-compulsion, interpersonal sensitivity, depression and paranoid ideation (Ji *et al*. 2017).

In the 2014 Middle Eastern MERS-CoV infection, healthcare workers experienced ‘emotional turmoil’ during their duty (Khalid *et al*. 2016: 8). Similarly with the severe acute respiratory syndrome (SARS) outbreak in China (Ji *et al*. 2017), 89% of healthcare workers in high-risk situations reported psychological symptoms (Chua *et al*. 2004). Nurses experienced fear of dying, feelings of isolation, loneliness, need for support and fear of exposing family members to the virus. Healthcare workers on the front line of the SARS outbreak were at risk of developing psychological distress. The psychological distress for SARS survivors appeared to be ‘substantial, pervasive and long-lasting’ (Gardner and Moallef, 2015: 133).

In the current COVID-19 outbreak in China, a significant proportion of healthcare workers experienced anxiety, depression and insomnia symptoms. More than 70% reported psychological distress. Specifically women, nurses and front-line healthcare workers had a high risk of developing unfavourable mental health outcomes and may require psychological support or intervention (Lai *et al*. 2020).

As COVID-19 spreads globally, healthcare workers are increasingly vulnerable to psychological distress. Government and healthcare systems have a responsibility to develop a range of supports to address this. Good management, feeling safe at work generally, having supportive colleagues and a positive atmosphere are also necessary components (Khalid *et al*. 2016). Drawing on our clinical experience and mindfulness practice, this paper will present a mindfulness tool that may be used by clinicians on the job.

## Mindfulness in clinical settings

Mindfulness can be defined as the awareness that emerges through paying attention on purpose, in the present moment and non-judgmentally to the unfolding of experience moment by moment (Kabat-Zinn, 2003). Mindfulness training for healthcare staff can promote self-care and well-being (Irving *et al*. 2009). Increases in self-compassion as a result of mindfulness programmes are particularly relevant to professional caregivers (Shapiro *et al*. 2007). Compassion for both self and others in a clinical setting is a necessary component to facilitating a therapeutic environment (Gilbert, 2005). Research demonstrates that by being compassionate and kind to ourselves and others we can help settle our feelings. ‘We can work on whatever problems we need to work on – from an understanding, kind and compassionate position’ (Gilbert, 2010: 31).

Growing research evidence shows the benefits of mindfulness on healthcare worker’s life satisfaction (Lomas *et al*. 2018), resilience to stress (Kinser *et al*. 2016), professional quality of life (Keogh *et al*. 2019), self-compassion (Wasson *et al*. 2020), trait mindfulness, compassion and general well-being (Ducar *et al*. 2020). The impact of a modified version of mindfulness-based stress reduction (MBSR) for emergency healthcare workers was specifically explored (Ducar *et al.* 2020 *)*.

Mindfulness is the foundation of self-compassion insofar as we can only respond self-compassionately when we know we are struggling (McGehee *et al*. 2017). Self-compassionate people react to adverse events in a more emotionally regulated pattern. Their responses are less extreme with less focus on negative affect and with more self-acceptance and an internal locus of control (Leary *et al*. 2007). Self-compassion is associated with a range of psychological strengths such as resilience, happiness, optimism, wisdom, curiosity, courage, exploration and emotional intelligence (Germer and Neff, 2013). Often people who are self-critical believe that without their inner critic, they will no longer be motivated to achieve their goals. However, research indicates that self-compassion can enhance motivation towards goal achievement and can facilitate soothing of maladaptive perfectionism (Neff, 2003).

A mindful self-compassion programme called Cultivate Your Inner Resilience (CYIR) was adapted from the Mindful Self-Compassion (MSC) programme (Neff *et al*. 2013) for healthcare workers in Ireland (Keogh *et al*. 2019). Significant improvements were found in stress, secondary traumatic stress and self-compassion immediately following the intervention. At a 6-month follow-up, self-compassion improvement had maintained. People with self-compassionate qualities are less self-critical and less likely to suffer with mental health issues (Beaumont *et al.* 2016). They have greater emotional regulation and resilience, are more capable of coping with stress and are less at risk of compassion fatigue and burnout (Thompson *et al*. 2014).

## Mindfulness moments for clinicians

Based on the clinical experience of the authors, we have highlighted several mindfulness practices that may be utilised by clinicians. These mindfulness moments for clinicians (MMFC) have been highlighted in supplementary Tables 1 and 2. MMFC may be used to support clinicians and promote resilience in the midst of a pandemic. All of our chosen practices are short and can be easily used by both frequent meditators and those who are new to the practice. Most importantly, they can be used ‘on the job’. MMFC incorporates evidence-based mindfulness practices for healthcare workers. Most of the practices have been extracted directly from the following evidence-based 8-week programmes: MSC (Neff *et al*. 2013), MBSR (Kabat-Zinn, 1990) and mindfulness-based cognitive therapy (MBCT) programmes (Segal *et al*. 2002). The RAIN (Recognise, Allow, Investigate and Nurture) practice stems from the work of mediation teacher Tara Brach (Brach, 2020). She refers to it as the ‘*RAIN of Self-Compassion’*. The acronym was first coined about 20 years ago by Michele McDonald. The GRACE practice has five elements: (1) Gathering attention: focus, grounding, balance. (2) Recalling intention: the resource of motivation. (3) Attuning to self/other: affective resonance. (4) Considering: what will serve. (5) Engaging: ethical enactment, then ending. Joan Halifax, a Buddhist teacher and anthropologist, developed the practice from her work on compassion in clinical care settings (Halifax, 2012). Gratitude meditation is strongly related to well-being (Emmons *et al*. 2003; Wood *et al*. 2010; O’Leary *et al.* 2015; Sirois *et al*. 2017). We have provided a gratitude practice by Tara Brach (Brach, 2017). Savouring meditation can greatly increase happiness and life satisfaction (Bryant & Veroff, 2007). The savouring practice provided is from the Greater Good Science Centre at the University of California, Berkeley.

To use the MMFC (supplementary Table 1), click the hyperlink to listen to or read the practice. See supplementary Table 2 for the authors’ modified version of these practices.

## Conclusion

Healthcare workers are at risk of psychological distress and burnout during their routine work. During this unprecedented pandemic, there is potential for this to be significantly exacerbated. Mindfulness has been shown to improve healthcare worker’s life satisfaction, resilience to stress, professional quality of life, self-compassion, trait mindfulness, compassion and general well-being. MMFC (supplementary Table 1) practices have the potential to support clinicians whenever they feel overwhelmed at work. These are short practices and easy to remember. By paying attention and noticing how they feel physically and psychologically, clinicians will be better able to recognise and meet their own needs. Compassion and self-compassion within the MFFC can also give comfort to the body and the mind affirming that courage is not absence of fear, but the willingness to be present and respond with equanimity in spite of it. MMFC is a simple and straightforward tool that may be easily incorporated into staff support training. Whilst the research indicates that mindfulness can help psychological distress in healthcare workers, it is only one tool. Governments and organisations have a responsibility to provide a range of other supports for healthcare workers. Further research is warranted to determine the effectiveness of using MMFC as it could potentially provide a pathway for clinicians to deepen their self-care through awareness of their body and mental state when providing care to their patients.

## Supplementary material

For supplementary material accompanying this paper visit http://dx.doi.org/10.1017/ipm.2020.59. click here to view supplementary material
