# Supplementary material for: Mindfulness moments for clinicians in the midst of a pandemic
Source: Ir J Psychol Med. 2020 May 21:1–4. doi: 10.1017/ipm.2020.59 (PMC7276502; doi:10.1017/ipm.2020.59)
Supplement: Supplementary file 1 [file S0790966720000592sup.zip › S0790966720000592sup002.docx]

**Supplementary Table 1.** Mindfulness moments for clinicians (MMFC).

| Practice | When |
| --- | --- |
| Affectionate breathing*  <https://self-compassion.org/wp-content/uploads/meditations/affectionatebreathing.mp3> | Feeling distressed, anxious, angry or sad. |
| Compassionate body scan*  <https://self-compassion.org/wp-content/uploads/meditations/bodyscan.MP3> | Experiencing physical or emotional discomfort. |
| Compassion with Equanimity*  <https://chrisgermer.com/wp-content/uploads/2017/09/Compassion-with-Equanimity-Germer.m4a> | Caring for a patient whilst feeling empathic distress and fatigue. |
| Compassionate words to self*  <https://center4msc-wpengine.netdna-ssl.com/wp-content/uploads/2016/03/Compassionate-Letter-Myself.pdf> | Experiencing negative emotions e.g. low mood, despondency and anxiety. |
| G.R.A.C.E**  <https://www.huffpost.com/entry/compassion-_b_1885877> | In a stressful clinical situation. |
| Gratitude***  <https://www.youtube.com/watch?v=g73ykQkGCnI> | Experiencing negative emotions e.g. low mood, despondency and anxiety. |
| R.A.I. N***  <https://www.tarabrach.com/meditation-the-rain-of-self-compassion/> | Feeling insecure or unworthy. |
| Savouring****  <https://ggia.berkeley.edu/practice/savoring_walk> | Experiencing negative emotions e.g. low mood, despondency and anxiety. |
| Self -compassion break*  <https://chrisgermer.com/wp-content/uploads/2017/04/Self-Compassion-Break-13-min.mp3> | Feeling distressed and/or coping with a difficult situation. |
| Soles of the feet*  <https://center4msc-wpengine.netdna-ssl.com/wp-content/uploads/2016/03/Soles-of-the-Feet_2017.pdf> | Feeling emotionally overwhelmed and needing to ground oneself. |
| 3 minute breathing Space*****  <https://www.youtube.com/watch?v=rOne1P0TKL8> | Feeling emotionally overwhelmed and needing to ground oneself. |

* MSC (Neff *et al*. 2013) and Center for Mindful Self-Compassion.

**Halifax (2012).

***Brach (2017)

***Brach (2015)

**** Greater Good Science Center.

*****MBSR (Kabat-Zinn, 1990) and MBCT (Segal et al. 2002).
