# Supplementary material for: Mindfulness moments for clinicians in the midst of a pandemic
Source: Ir J Psychol Med. 2020 May 21:1–4. doi: 10.1017/ipm.2020.59 (PMC7276502; doi:10.1017/ipm.2020.59)
Supplement: Supplementary file 1 [file S0790966720000592sup.zip › S0790966720000592sup001.docx]

**Supplementary Table 2.** Mindfulness moments for clinicians (MMFC): modified practices.

Starting all these practices with a few slow, easy breaths releasing any unnecessary tension. Lowering the gaze or closing the eyes.

| Practice | Modified version (adapted by the authors) |
| --- | --- |
| Affectionate breathing. | Feeling the inbreath nourishing the body and noticing how the body relaxes with the outbreath. Feeling the rhythm and movement of the breathing. |
| Compassionate body scan. | Starting with the toes on your left foot and noticing if there are any sensations in your toes. Perhaps giving your toes an inner smile of recognition or appreciation. With the same sense of interest, curiosity and kindness, moving your attention slowly throughout the body. Being kind, appreciative, compassionate and respectful to the body. |
| Compassion with Equanimity. | Bringing to mind the person you are caring for - visualising both of you. Feeling the struggle in your body. Repeating the following words: ‘I am not the cause of this person’s suffering, nor is it entirely within my power to make it go away. Moments like this are hard to bear, yet I may still try to help if I can’. Inhale fully and deeply, drawing compassion inside your body. As you exhale, sending compassion to this other person. Continue breathing compassion in and out. ‘In for me and out for you’. |
| Compassionate words to self. | In your mind, speak to a dearly loved friend who is struggling with the same concerns as you. Now say those words to yourself. Alternatively think of an imaginary friend who is unconditionally wise, loving and compassionate. Speak to yourself with his/her voice. |
| G.R.A.C.E. | Gather your attention by pausing and focusing on the breath. Recall your intention by remembering why you have chosen to relieve the suffering of others and to work in this way. Attune to yourself and your patient by noticing and reflecting on what you are both thinking, feeling and sensing right now. Consider what is needed. Engage, enact ethically and end by breathing out to explicitly recognize internally that the encounter is over. |
| Gratitude. | List 3 things you are grateful for in your life. They may be relatively insignificant things you usually overlook such as buttons, warm water or a genuine smile. |
| R.A.I. N. | Recognize what is happening by acknowledging the thoughts, feelings, and behaviours that are affecting you. Allowing them simply to be there, without trying to fix or avoid anything. Investigate your experience with curiosity and kindness. Nurture your vulnerable self. |
| Savouring. | Recognise any pleasant experience that occurs in your day, no matter how small. Allow yourself to be drawn to it. Linger with it. Let it go. |
| Self-compassion break. | Beginning to sense into your body. Acknowledging that you are experiencing some emotional pain/stress. Reminding yourself that difficult experiences are part of life. Deciding to be kind to yourself because you are struggling right now. |
| Soles of the feet. | Noticing the sensations in the soles of your feet where they connect to the ground.  Each time the mind wanders, just come back to feeling the soles of the feet again. |
| 3 Minute Breathing Space. | Recognising and acknowledging your current thoughts, feelings and physical sensations. Bringing your attention to the sensations of the breath in the body. Slowly expanding this awareness into the body as a whole. |
